# Glycolysis: A multifaceted metabolic pathway and signaling hub

**DOI:** 10.1016/j.jbc.2024.107906

**Published:** 2024-10-22

**Authors:** Sarah J. Kierans, Cormac T. Taylor

**Affiliations:** 1 Conway Institute of Biomolecular and Biomedical Research, University College Dublin, Dublin, Ireland; 2 UCD School of Medicine, University College Dublin, Dublin, Ireland

**Keywords:** glycolysis, cellular signaling, cellular metabolism, bioenergetics, lactate, ATP, metabolon

## Abstract

Glycolysis is a highly conserved metabolic pathway responsible for the anaerobic production of adenosine triphosphate (ATP) from the breakdown of glucose molecules. While serving as a primary metabolic pathway in prokaryotes, glycolysis is also utilized by respiring eukaryotic cells, providing pyruvate to fuel oxidative metabolism. Furthermore, glycolysis is the primary source of ATP production in multiple cellular states (*e.g*., hypoxia) and is particularly important in maintaining bioenergetic homeostasis in the most abundant cell type in the human body, the erythrocyte. Beyond its role in ATP production, glycolysis also functions as a signaling hub, producing several metabolic intermediates which serve roles in both signaling and metabolic processes. These signals emanating from the glycolytic pathway can profoundly impact cell function, phenotype, and fate and have previously been overlooked. In this review, we will discuss the role of the glycolytic pathway as a source of signaling molecules in eukaryotic cells, emphasizing the newfound recognition of glycolysis’ multifaceted nature and its importance in maintaining cellular homeostasis, beyond its traditional role in ATP synthesis.

## Glycolysis and its contribution to metabolism

The glycolytic pathway is a highly conserved metabolic process responsible for the anaerobic catabolism of glucose with the concomitant production of ATP. The pathway consists of 10 metabolic enzymes which work in a sequential manner to convert a six-carbon glucose molecule into two-, three-carbon molecules of the tricarboxylic acid cycle precursor, pyruvate. This anaerobic conversion occurs within the cytoplasm and results in a net gain of two molecules of ATP, two molecules of reduced nicotinamide adenosine dinucleotide (NADH), and two molecules of water per molecule of glucose entering the pathway. Under physiologic oxygen (O_2_) tensions, the pyruvate generated from glycolysis is transported into the mitochondria where it undergoes O_2_-dependent oxidation to form acetyl coenzyme A (acetyl co-A). This acetyl co-A is metabolized by the tricarboxylic acid cycle and the electron transport chain during oxidative phosphorylation to produce a significantly greater amount of ATP per glucose molecule than produced from glycolysis alone.

While glycolysis serves as the initial step in cellular respiration in most respiring cells, it plays a crucial role as the primary source of ATP in cells lacking mitochondria (*e.g.,* erythrocytes) or in cells experiencing hypoxia. Under such conditions, a significant increase in glycolytic flux is essential to maintain ATP production and bioenergetic homeostasis when mitochondrial metabolism is reduced or absent. This increased flux is supported by the fermentation of pyruvate into lactate, a process which concurrently regenerates nicotinamide adenine dinucleotide (NAD^+^), an essential cofactor for sustained glycolytic flux, from its reduced form, NADH. Cellular respiration, encompassing both glycolysis and mitochondrial respiration, is a highly regulated process. This precise regulation allows for efficient energy production, while permitting rapid adjustments to the rate of respiration to be made to ensure the dynamic energy requirements of the cell in a changing environment are met. While we have previously discussed the transcriptional control of glycolysis and its effects on cell metabolism (1), this review will focus on our current understanding of the role of the glycolytic pathway as a signaling source in eukaryotic cells.

## Spatial localization of glycolytic metabolism

Given the complexity of cellular metabolism, it is perhaps unsurprising that eukaryotic cells have evolved to partition their metabolic activities into discreet membrane-bound structures. These organelles, such as mitochondria and lysosomes, host distinct metabolic activities allowing for complex biochemical reactions to take place under optimal conditions and with a high degree of spatial organization. While this compartmentalization of enzymes involved in a given metabolic pathway is biologically advantageous, glycolytic enzymes have historically been considered to be soluble proteins freely dispersed in the cytosol of eukaryotic cells. This view, which presented glycolysis as a simple series of enzymatic reactions occurring in a homogenous cytosolic environment, remained unchallenged until the late 20th century. Subsequent discoveries revealed that glycolytic enzymes are in fact ambiquitous molecules, capable of dynamically partitioning between soluble and particulate bound states, depending on cytosolic conditions (2, 3, 4). The dual nature of glycolytic enzymes is now supported by a large body of cross-kingdom evidence demonstrative of their ability to exist as freely soluble enzymes and as components bound to subcellular structures, such as muscle fibers, organelles, and the plasma membrane (5, 6, 7, 8, 9, 10, 11, 12, 13, 14, 15, 16).

The characterization of the dual nature of glycolytic enzymes prompted the hypothesis that glycolytic enzymes can become organized into a functional complex or ‘metabolon’ as means to optimize glucose metabolism (17). Substantial cross-kingdom evidence now supports the existence of glycolytic complexes in plants (*Arabidopsis thaliana)* (18, 19), yeast (*Saccharomyces cerevisiae*) (20, 21), nematodes (*Caenorhabditis elegans*) (22), and mammalian cells (23, 24, 25). To date, the strongest evidence for the formation and metabolic benefit of such complexes comes from the parasitic protist *Trypanosoma brucei*, where 9 of the 10 glycolytic enzymes are located within membrane-bound organelles termed glycosomes (26). This compartmentalization of glycolytic enzymes in intact glycosome is crucial for the survival of the protozoan parasite *T. brucei* (27), particularly during periods of anaerobiosis.

The organization of metabolic enzymes into supramolecular complexes, whether membrane bound or not, can be understood from both metabolic and regulatory perspectives. A key benefit of metabolon formation is in the facilitation of substrate channeling, *i.e.,* the direct transfer of an intermediary reaction product from one enzyme’s active site, to the next, bypassing the need for the substrate to equilibrate in the bulk solution of the cytoplasm (28). In the context of glycolysis, however, channeling of intermediates is unlikely to increase the steady-state reactions of glycolytic enzymes, as diffusion is not typically a limiting factor to the rate of reactions (triose phosphate isomerase being an exception to this with a sufficiently high K_cat_/K_M_ ratio) (29). Instead, metabolon formation here may play an important role in limiting the diffusion of intermediates to competing metabolic pathways or alternatively contribute to the reduction of a high cytoplasmic viscosity which may hamper biological processes (30). The identification that glycolytic enzymes have the propensity to colocalize with one another importantly highlights the formation of intracellular microenvironments where gradients of intermediates or products can be established between different enzymatic active sites within the complex (17).

Of note, the formation of such metabolic complexes is not an invariant property of the cell. Rather, these aggregates form transiently in a manner often contingent on the specific requirements of a cell, tissue, or organism. Glycolytic complex formation has been described in response to hypoxic stress (20, 21, 23), elevated respiratory demand (31), or nutrient deprivation (32), suggesting that alterations in the spatial and temporal organization of glycolysis could be an important adaptive response to cellular stress. In the yeast *S. cerevisiae*, disruption of glycolytic complex formation confers a competitive growth disadvantage relative to cells where glycolytic complexes remain intact (21). In the nematode *C. elegans,* synaptic transmission is impaired when glycolytic complex formation is inhibited (22), while impaired glycolytic complex formation in the flight muscle of *Drosophila melanogaster* renders the fruit fly unable to take flight (33). Each of these functional consequences arise despite a full complement of glycolytic enzyme expression in these species, thereby demonstrating the necessity for spatiotemporal organization of the pathway.

## Products of glycolysis in cellular signaling

The formation of complexes involved in glucose metabolism raises important questions regarding how these structures may create localized gradients of substrates, products, and regulatory molecules, and how these gradients may influence the regulation of cellular processes and signaling pathways. Recent research has revealed that in addition to the nonglycolytic or moonlighting functions of glycolytic enzymes (34, 35, 36), glycolytic intermediates also serve as important signaling molecules, influencing a wide range of cellular processes beyond the provision of energy. This newfound recognition underscores the multifaceted nature of glycolysis and emphasizes its significance in maintaining cellular homeostasis.

### Glycolytic intermediates

**Figure 1:**
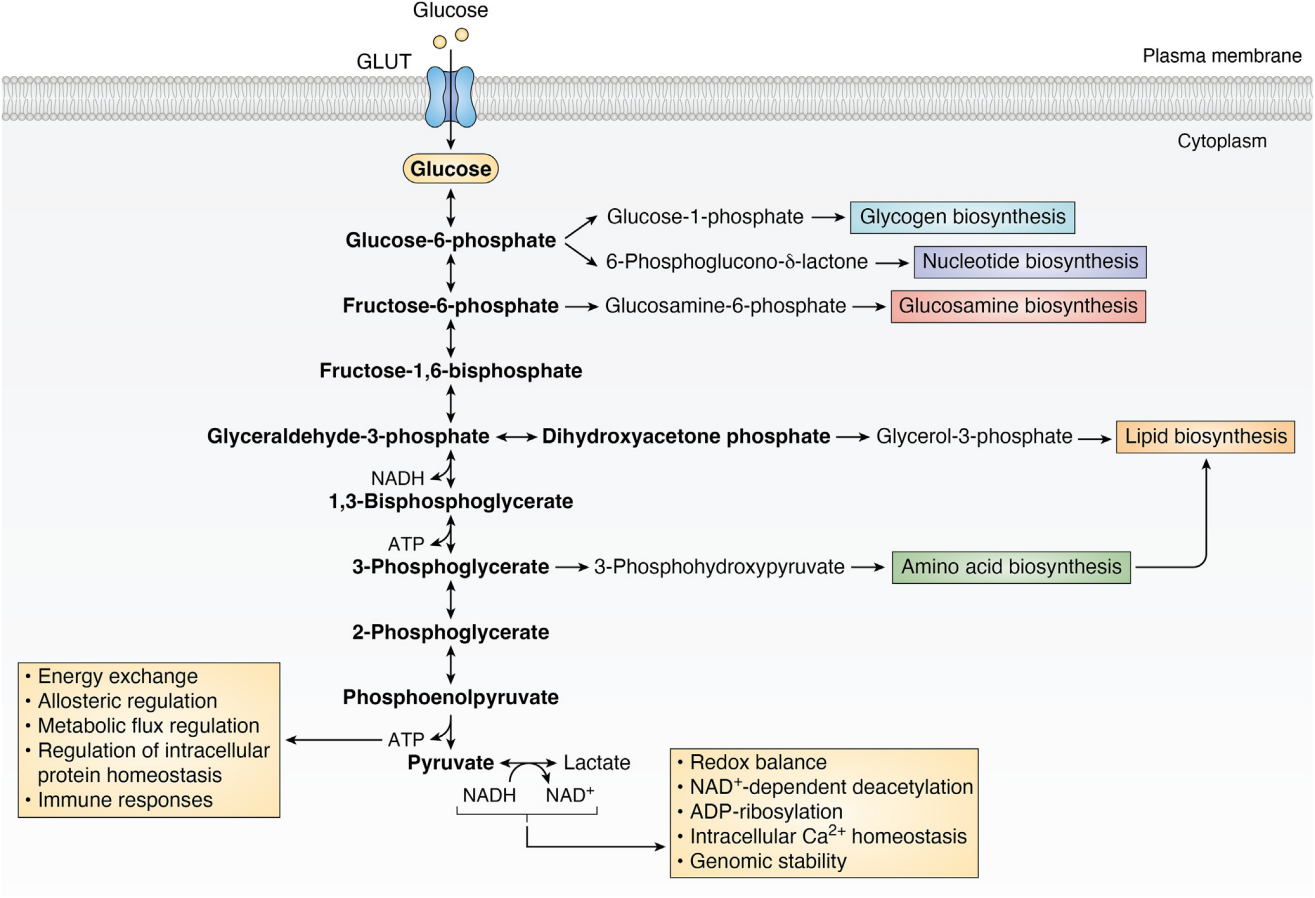
**Glycolytic products and intermediates influence a wide range of cellular processes, beyond the provision of energy.** Schematic outlining the various roles of glycolytic products, ATP and NADH, and glycolytic intermediates (bold text), beyond their roles in energy production. These roles include the synthesis of biomolecules essential for cellular structure, function, redox balance, and the regulation of immune responses, for example. NAD^+^, nicotinamide adenine dinucleotide; NADH, nicotinamide adenosine dinucleotide.

In addition to its role as a central pathway for ATP production, glycolysis produces multiple intermediate products important for a various anabolic reactions within cells (summarized in Fig. 1). For instance, the glycolytic intermediate glucose-6-phosphate can be diverted into the pentose phosphate pathway, an important anabolic pathway responsible for generating ribose for nucleotide biosynthesis and reductive power in the form of nicotinamide adenine dinucleotide phosphate for protection against cellular oxidative stress (37). The glycolytic intermediate fructose-6-phosphate serves a key substrate for the hexosamine biosynthetic pathway (38), while dihydroxyacetone phosphate acts as a precursor to glycerol-3-phosphate—an essential intermediate in the biosynthesis of major structural phospholipids and triacylglycerols. Moreover, the glycolytic intermediate 3-phosphoglycerate contributes carbons essential for synthesizing sphingolipids and amino acids serine, cysteine, and glycine (37). While these glycolytic intermediates facilitate anabolic reactions within cells to support cell growth and proliferation, the diversion of glucose-derived carbons into pathways emanating from glycolysis can have detrimental effects in pathophysiological conditions such as cancer development and progression (37).

While glycolytic intermediates have been traditionally viewed solely as metabolic products and substrates, several glycolytic intermediates have also emerged as important signaling molecules in diverse cellular responses. For example, phosphoenolpyruvate can modulate T cell effector function by sustaining T cell receptor–mediated Ca^2+^-nuclear factor of activated T cells signaling, promoting a proinflammatory phenotype (39). Phosphoenolpyruvate also regulates T cell responses by inhibiting Th17 cell differentiation and the production of Th17/Th2 cell cytokines (40). Other intermediates, such as fructose-1,6-bisphosphate, exhibit neuroprotective effects by suppressing Toll-like receptor 4 signaling in the brain, thereby reducing neuroinflammation (41). The glycolytic intermediates glucose-6-phosphate, fructose-6-phosphate, fructose-1,6-bisphosphate, and 1,3-bisphosphoglycerate link glucose metabolism with excitation–contraction coupling in the heart *via* their regulation of intracellular Ca^2+^ release (42, 43) and their activation of sarcolemmal ATP-sensitive K^+^ channels (44). Dihydroxyacetone phosphate signals glucose availability to mammalian target of rapamycin complex 1, independent of intracellular adenosine nucleotide sensor, adenosine monophosphate–activated protein kinase (AMPK) (45). This activation of mammalian target of rapamycin complex 1 can then promote glycolysis *via* a HIF-1-driven transcriptional upregulation of pathway components to sustain glycolytic flux (46). Finally, the glycolytic enzyme GAPDH plays a crucial role in cellular tolerance to oxidative damage (47). Inhibition of GAPDH through oxidation (47), NADH reductive stress (48, 49, 50), or specific inhibitors (51), results in rerouting of glucose-derived carbons through the oxidative branch of the pentose phosphate pathway. This enables cells to better regulate their nicotinamide adenine dinucleotide phosphate levels, thereby enhancing their ability to withstand redox stress.

Collectively, these findings demonstrate that glycolysis serves not only as a central pathway for energy production but also as a source of intermediates essential for supporting various anabolic reactions and signaling processes within the cell necessary to sustain cell growth and proliferation.

### Lactate

**Figure 2:**
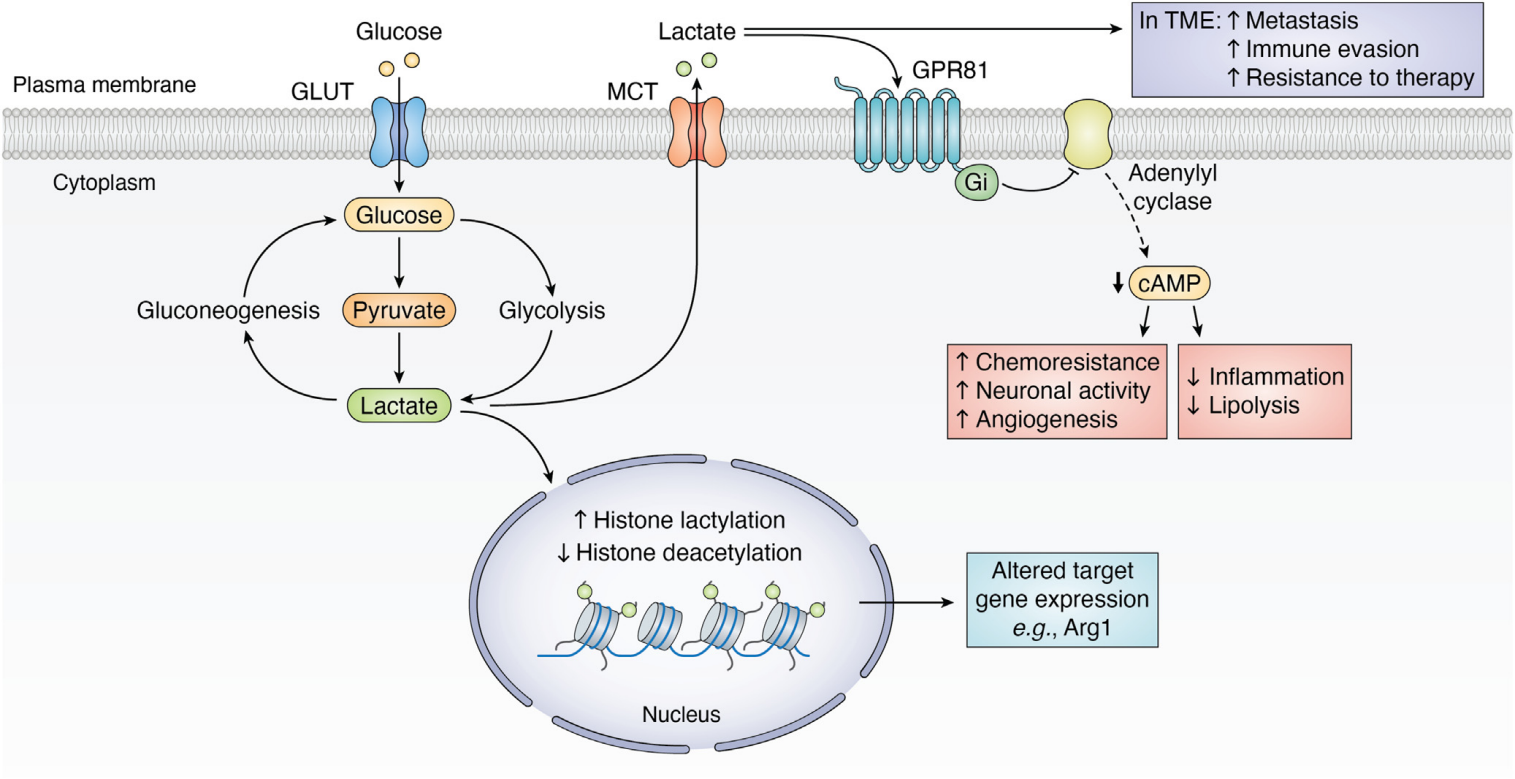
**Lactate as a pleiotropic signaling molecule.** Schematic summarizing the various roles of lactate including its role as a major gluconeogenic precursor, a regulator of gene expression *via* lactylation of histones, and a signaling molecule with both autocrine and paracrine effects. MCT, monocarboxylate transport protein.

Historically, the glycolytic end-product lactate has been regarded as a waste product of anaerobic glucose metabolism without any discernible biological function (52). In more recent years, lactate has gained attraction as a pleiotropic molecule, playing a significant role in intracellular metabolism and energy homeostasis (summarized in Fig. 2). In exercising skeletal muscle (53, 54, 55), heart muscle (53, 56, 57, 58, 59), the brain (60, 61, 62, 63, 64), and cancerous cells (57, 65, 66), lactate is preferred over glucose as an oxidative fuel source. Moreover, circulating lactate plays a metabolically beneficial role as a primary gluconeogenic precursor (67), contributing to the maintenance of blood glucose levels during intense exercise or fasting (68, 69).

While lactate accumulates acutely during intense exercise, approximately 50% of circulating lactate is oxidized at rest, increasing to as much as 80% during exercise (53, 54, 55, 70). Lactate which has not been directly oxidized can be transported across biological membranes by either proton-coupled (71) or sodium-coupled monocarboxylate transport proteins (72). When released into the systemic circulation, lactate serves as a pleiotropic signaling molecule. Lactate is an endogenous ligand for GPR81 (hydroxycarboxylic acid receptor 1 or HCAR1), a G_i_ protein-coupled receptor predominately expressed in adipose tissue (73, 74). Binding of lactate to GPR81 mediates an antilipolytic effect through the Gα_i_-dependent inhibition of adenylyl cyclase and the cyclic adenosine monophosphate–protein kinase A pathway (75, 76). While predominately expressed in adipose tissue, GPR81 is also expressed in immune cells, in the brain, in skeletal muscle, and within tumors, where lactate can induce nonmetabolic functional effects. Activation of the lactate-GPR81 axis in colonic dendritic cells and macrophages plays an important role in reducing colonic inflammation through suppression of proinflammatory cytokine release (77). Lactate further negatively regulates the Toll-like receptor induction of the NLRP3 inflammasome, nuclear factor-κB, and interleukin-1β release from macrophages *via* β-arrestin 2 (78), in addition to suppressing macrophage proinflammatory responses for protection against inflammation (79, 80). In the brain, lactate-induced signaling through GPR81 is linked to neuronal activity (81, 82), neuroprotection (83), and neurogenesis (84, 85). Finally, in the context of cancer, GPR81 expression is positively correlated with the rate of tumor growth and metastasis *in vivo* (86) with lactate signaling through GPR81 having been shown to promote angiogenesis (87), chemoresistance (88), and antitumor immunity (89, 90).

Lactate’s role as a signaling molecule however is not limited to its actions mediated through GPR81. Research has recently highlighted a role for the enantiomer L-lactate in epigenetic gene regulation through the posttranslational modification of histone lysine residues (lactylation) (91). This dynamic posttranslational modification is sensitive to both exogenous and endogenous lactate levels (92) and affects epigenetic gene regulation by stimulating gene expression from chromatin (91). Lactylation has thus far been identified to have important implications for macrophage polarization (91, 93), microglial activation (94), tumorigenesis (95, 96), cancer progression (97), DNA repair, and chemoresistance (98). Lactate can also further alter the epigenome *via* inhibition of histone deacetylases to promote further changes in gene expression (99).

Finally, while lactate accumulates acutely during intense exercise, its accumulation becomes prolonged in inflamed niches or within the tumor microenvironment. High concentrations of lactate in solid tumors are commonly associated with metastasis (100, 101, 102, 103), immune evasion (104, 105, 106, 107, 108), and resistance to therapy (109, 110), through modulation of the inflammatory response in Th17 cells (111), macrophages (79, 112), and monocytes (78, 113) and lactate-induced reductive stress–dependent inhibition of T cell proliferation (48).

Taken together, these findings indicate that lactate is not merely a byproduct of glycolytic metabolism but instead plays significant roles in regulating various physiological and pathophysiological processes such as inflammation, immunity, and tumor progression.

### ATP

Glycolysis produces a net gain of two molecules of ATP per molecule of glucose. ATP is predominately recognized for its role as a universal energy carrier in cells, providing energy for various biochemical reactions such as muscle contraction, nerve impulse propagation, and active transport. Beyond its role in energy exchange, ATP plays dual roles in maintaining energy homeostasis. ATP acts as an allosteric inhibitor of rate-limiting enzymes of glycolysis, phosphofructokinase-1, and pyruvate kinase, reducing the rate of glycolysis when a cell’s energy charge is high (114). Additionally, ATP plays a crucial role in regulating the activity of AMPK, a heterotrimeric αβγ complex that acts as a key regulator of cellular energy homeostasis. ATP competitively inhibits the binding of AMP or ADP to the γ subunit of AMPK, thus inhibiting its activation (115, 116, 117). Conversely, when ATP levels are depleted—due to increased ATP consumption (*e.g.,* during exercise) or decreased ATP generation (*e.g.,* during hypoxia)—the reduction in ATP:AMP and ATP:ADP ratios allows AMP/ADP to bind AMPK’s regulatory subunit. AMP/ADP binding promotes AMPK phosphorylation and activation by upstream kinases while also protecting against dephosphorylation and deactivation by phosphatases (117, 118). Furthermore, AMP binding results in allosteric activation, enhancing AMPKs ability to respond to cellular energy deficits and restore bioenergetic homeostasis (119, 120, 121). Once activated, AMPK regulates energy balance by activating catabolic pathways responsible for ATP production and inhibiting anabolic processes which consume ATP (122). For instance, AMPK increases glycolytic flux by phosphorylation and activation of phosphofructokinase-2 (123, 124) while suppressing fatty acid and sterol synthesis by inhibiting key enzymes such as acetyl co-A carboxylase and HMG-CoA reductase (125).

ATP has also emerged as a versatile regulator of protein homeostasis, acting as a biological hydrotrope within intracellular compartments (126). As an amphiphilic compound with both hydrophilic and hydrophobic properties, ATP can solubilize hydrophobic protein aggregates and prevent pathologic protein aggregation in an energy-independent manner (126). ATP has the propensity to influence protein solubility and liquid–liquid phase separation through its bivalent binding of its hydrophobic adenine moiety to charged amino acids within intrinsically disordered regions of multiple proteins (127, 128, 129, 130). ATP has also been shown to play a pertinent role in inducing protein folding (131, 132), suggesting an important role for the energy carrier in regulating the material properties of the cell interior. Notably, the biphasic regulation of liquid–liquid phase separation by ATP, and the contribution of ATP to protein folding occurs at mM concentrations of ATP, which could explain why intracellular concentrations of ATP range from 2 to 10 mM (depending on cell type) despite all known ATP-dependent proteins/enzymes requiring only micromolar concentrations of ATP (133).

In addition to its intracellular roles, ATP also functions as a ubiquitous extracellular messenger. In response to inflammation (134), hypoxia (135, 136), hypercapnia (135), osmotic pressure (137), sheer stress (134, 138), or cell damage, ATP can be released extracellularly where it initiates responses through activation of nucleotide-responsive purinergic P2 receptors located on the cell surface. ATP-responsive purinergic signaling controls several aspects of various physiological and pathophysiologic processes, including neurotransmission (139) and the regulation of immunity. ATP released from apoptotic or damaged cells, for example, promotes the ATP receptor (P2Y2)–dependent recruitment of monocytes and macrophages to promote phagocytic clearance (140). At sites of inflammation, ATP released from immune cells can act in an autocrine manner to alter leukocyte function, promoting macrophage polarization (141), T-cell activation (142, 143), and neutrophil chemotaxis (144) in order to enhance bacterial clearance. Extracellular ATP can also act in a paracrine manner *via* purinergic receptors to modulate B cell (145) and T cell activation (146, 147), as well as dendritic cell activation, maturation, and antigen presentation (148, 149, 150). Signaling of ATP through purinergic receptors on immune cells can therefore elicit both positive and negative feedback mechanisms in the regulation of an immune response.

In summary, ATP serves not only as the primary energy currency for biochemical reactions but also mediates diverse roles both intracellularly and extracellularly (summarized in Fig. 1). These roles including the regulation of inflammation and immunity, the material properties of the cell, and kinase activation underscore ATP’s importance in maintaining cellular health and homeostasis.

### NADH and NAD^+^

During glycolysis, NADH is generated when glyceraldehyde-3-phosphate is oxidized to 1,3-bisphosphoglycerate. Both NADH and its oxidized form, NAD^+^, play essential roles in maintaining redox balance (NAD^+^/NADH), ensuring that key metabolic pathways, such as glycolysis and oxidative phosphorylation, proceed efficiently by facilitating redox reactions. Beyond their established roles in redox reactions, however, NAD^+^ and NADH also play critical roles in a variety of cellular processes, many of which are mediated by NAD^+^-consuming enzymes. For example, NAD^+^ is an essential substrate for the sirtuin family of deacetylases (151). Sirtuins are involved in a variety of biological processes, including the regulation of metabolism (152) and inflammation (153), as well as promoting cellular longevity by deacetylating lysine residues on histones and other proteins, thereby modifying gene expression (154, 155, 156, 157). Importantly, while dependent on NAD^+^ for their activation, Sir2 in yeast (158) and the human homolog SIRT1 (159) are competitively inhibited by NADH, which leads to a shortened yeast lifespan and a reduced rate of cellular metabolism. This finding underscores the importance of the NAD^+^/NADH ratio as a key regulator of biological processes, such as longevity, in addition to its role as a marker of intracellular redox state. In addition to its role in facilitating sirtuin deacetylation, NAD^+^ also serves as a substrate for mono-ADP ribosyl transferases and poly-ADP ribosyl polymerases, the enzymes responsible for ADP-ribosylation (160). This reversible posttranslational modification plays critical roles in DNA repair (161, 162), gene transcription (163, 164, 165), and the regulation of intracellular signaling pathways, such as the nuclear factor kappa B and Erk-signaling pathways (166, 167). Given the pertinent role for NAD^+^-dependent enzymes in metabolism, DNA repair, and inflammatory processes, it is perhaps unsurprising that depletion in cellular NAD^+^ and a reduced NAD^+^/NADH ratio are associated with cellular senescence, aging, and the onset of age-related disease (168).

Furthermore, NAD^+^ can be used as a substrate for cyclic ADP-ribose synthases. Cyclic ADP-ribose synthases convert NAD^+^ into second-messengers cyclic ADP-ribose or ADP-ribose which regulate Ca^2+^ homeostasis through the mobilization of intracellular Ca^2+^ stores (169, 170, 171) or by modulating Ca^2+^ influx *via* TRPM2 (transient receptor potential melastatin-related) cation channels (172). Within the nucleus, NAD^+^ and NADH also modulate gene expression by regulating 5′ RNA capping, an important modulator of RNA stability and gene expression (173, 174, 175), while NADH has also been shown to modulate gene expression by promoting the DNA-binding activity of the heterodimers important in circadian clock regulation (176) or potentiating carboxyl terminal–binding protein–mediated transcriptional repression (177).

Therefore, maintaining an appropriate NAD^+^/NADH ratio, both intracellularly and within distinct intracellular compartments, is not only essential for regulating ATP synthesis but also further plays a pivotal role in diverse biological processes such as genomic stability, Ca^2+^ homeostasis, and aging (summarized in Fig. 1).

## Conclusion

Glycolysis, an ancient and essential metabolic pathway, is now recognized as a key cellular signaling hub with functions extending far beyond its established role in glucose metabolism. While research has previously focused on the transcriptional control of glycolysis and its effects on cell metabolism, the nonglycolytic signaling roles for glycolytic intermediates and products described here rely more on the allosteric regulation and spatial organization of the pathway. We hypothesize that the colocalization of glycolytic enzymes into metabolic complexes can act as important signaling hubs which can profoundly impact cell function, phenotype, and fate. However, many questions remain regarding these complexes, including the temporal, qualitative, and quantitative nature of their formation and maintenance, as well as the mechanisms underpinning the formation of these interactions between constituent enzymes and probable scaffolds. Addressing these questions will not only enhance our understanding of the diverse roles of glycolysis in cellular signaling but may also highlight potential targets for therapeutic intervention in disease related to metabolic and signaling dysregulation.

## Conflict of interest

The authors declare that they have no conflicts of interest with the contents of this article.
